# Double-chamber syringe versus classic syringes for peripheral intravenous drug administration and catheter flushing: a study protocol for a randomised controlled trial

**DOI:** 10.1186/s13063-019-3887-1

**Published:** 2020-01-14

**Authors:** Pedro Parreira, Liliana B. Sousa, Inês A. Marques, Paulo Santos-Costa, Luciene M. Braga, Arménio Cruz, Anabela Salgueiro-Oliveira

**Affiliations:** 1 Health Sciences Research Unit: Nursing (UICISA:E), Nursing School of Coimbra (ESEnfC), Avenida Bissaya Barreto, Apartado 7001, 3046-851 Coimbra, Portugal; 2 Biophysics Institute, Coimbra Institute for Clinical and Biomedical Research (iCBR) area of CIMAGO, Faculty of Medicine, CNC.IBILI, Faculty of Medicine, University of Coimbra, Polo das Ciências da Saúde Azinhaga de Santa Comba, 3000-354 Coimbra, Portugal; 3 Departamento Medicina e Enfermagem, Universidade Federal Viçosa, Av. Peter Henry Rolfs, s/n Campus Universitário, Viçosa, MG 36570-900 Brazil

**Keywords:** Double-chamber syringe, Clinical research, Effectiveness, Safety

## Abstract

**Background:**

The prevention of catheter-related complications is nowadays an important topic of research. Flushing catheters is considered an important clinical procedure in preventing malfunction and several complications such as phlebitis or infection. Considering the latest guidelines of the Infusion Nurses Society, the flushing should be carried out both pre- and post-drug administration, requiring different syringes (with associated overall increased times of preparation/administration of intravenous medication by nurses, and also increasing the need for manipulation of the venous catheter).

**Methods/design:**

A multi-centre, two-arm randomised controlled trial with partially blinded outcome assessment of 146 adult patients. After eligibility analysis and informed consent, participants will receive usual intravenous administration drugs with flushing procedures, with a double-chamber syringe (arm A) or with classic syringes (arm B). The outcomes assessment will be performed on a daily basis by an unblinded ward team, with the same procedures in both groups. Some main outcomes, such as phlebitis and infiltration, will also be evaluated by nurses from a blinded research team and registered once a day.

**Discussion:**

The study outlined in this protocol will provide valuable insight regarding the effectiveness and safety of this new medical device. The development of this medical device (dual-chamber syringe, for drug and flush solution) seems to be an important step to facilitate nurses’ adoption of good clinical practices in intravenous procedures, reducing catheter manipulations.

**Trial registration:**

ClinicalTrials.gov, NCT04046770. Registered 13 August 2019.

## Background

The insertion of a peripheral intravenous catheter (PIVC) is the most frequent invasive procedure performed in nursing clinical practice. These invasive devices are inserted into patients’ peripheral veins and enable the intravenous administration of fluids, blood products, and drugs directly into the bloodstream [1, 2]. However, a wide range of complications can occur, such as mechanically induced complications (partial dislodgement or accidental removal, infiltration, extravasation, occlusion) [3–13], those of an infectious nature (bacterial or fungal sepsis) [7, 8, 10, 12, 14], or phlebitis [3–5, 15].

Phlebitis (irritation or inflammation of the vein wall, associated with warmth, tenderness, erythema, or palpable cord) is the most frequent PIVC-related complication, which may have mechanical, chemical, or bacterial causes [5, 13]. Thus, assessment of the PIVC insertion site must be performed daily and use of a transparent sterile dressing for stabilizing and protecting the catheter [16] is essential to allow for visual inspection of the catheter site. Transparent sterile dressings require less frequent changes than do standard gauze and tape dressings [17–19], and have also been associated with fewer accidental PIVC removals [20]. The main repercussion of phlebitis is pain, resulting in the need for new catheterization and momentary interruption of the prescribed intravenous therapy. Moreover, extravasation or infiltration of fluids may be responsible for local oedema due to the pervasion of intravenous fluid into the interstitial compartment, causing inflammation of the tissue around the catheter site. Occlusion is defined as any circumstance in which the PIVC is not able to be flushed or infuse fluids/medications and it is a clinical sign of catheter malfunction [21].

The use of a PIVC has also been associated with the risk of nosocomial bacteraemia [22], resulting in significant rates of patient morbidity and mortality [23, 24]. The European Centre for Diseases Control and Prevention (ECDC) indicates a higher infection prevalence in Portugal (10.6%) compared with the European mean prevalence (5.7%) [25]. In fact, the last report of the Program for Prevention and Control of Infection and Resistance to Antimicrobials (PPCIRA) for 2018 states an infection prevalence of 7.8% for acute hospitals in Portugal, one of the highest in Europe [26]. The prevention of catheter-related complications usually relies on precautions during catheter insertion (e.g. hand hygiene, use of the aseptic non-touch technique, catheter size, anatomical insertion site, dressings) and catheter surveillance (e.g. maintenance time, flushing, medications) and the overall competence and qualifications of the nurses [16–19, 27].

Flushing the catheters with 0.9% sodium chloride is the most important factor in preventing malfunction by maintaining catheter patency. International standards of care recommend the flush volume to be at least equal to twice the internal volume of the catheter system (catheter, extension set, and/or needleless injection system) [17]. In fact, professionals’ flushing practices appear to vary widely with regard to solutions, frequency, volumes, and techniques [13, 21, 28, 29]. PIVCs should be flushed at the time of medication administration [17, 30]. The flushing practice usually implies the initial aspiration of blood to ascertain vascular access device patency and minimum pre- and post-drug administration flushes using a pulsatile technique [17]. This has proved to be a simple, effective, and inexpensive technique to reduce catheter bacterial colonization [31]. The theoretical purpose of flushing is to maintain catheter patency by preventing internal luminal occlusion, reducing build-up of blood or other products on the device’s internal surface, and preventing interactions between fluids or drugs [32–36]. This procedure is also believed to prevent some important complications such as line blockages by blood clots or air bubbles/occlusion, phlebitis, and infection [37].

Traditionally, this process has been done using two or more syringes to assess patency (pre-flushing), drug delivery, and final flush solution. This process is time-consuming for nurses (both in the preparation and administration of the intravenous therapeutics), requires more manipulations of the venous access, and involves higher economic costs. Commercially prepared prefilled flush syringes are useful in reducing preparation and administration times as well as promote adherence to some of the recommended clinical practices, such as the aseptic non-touch technique and intravenous flushing practice [29]. Despite this, they do not address the need to reduce the number of catheter manipulations as well as the associated economic costs (using pre-filled syringes still dictates that the professional needs two or more syringes to accomplish the intravenous therapeutic process).

Double-chamber syringes have been developed as a drug and device combination product that enables the reconstitution and administration of drugs in fixed doses. Some of the existing syringes marketed for the administration of multiple fluids have not been well-received because they generally require the delivery of two fluids, one prefilled flush solution and an empty chamber to be filled with the medication. However, no syringe has become widely accepted that allows filling both chambers of a single syringe in place, one for routine medication administration and the other for subsequent catheter flushing. To address this gap in the market, a double-chamber syringe was developed that enables the filling and administration of drug and flush solution. The clinical effectiveness and safety parameters of this double-chamber syringe need to be determined.

## Methods/design

### Objectives

The main objective is to establish the clinical effectiveness and safety parameters of the new medical device: a double-chamber syringe for intravenous administration and flushing. In order to accomplish this objective, the main outcomes measured are related to PIVC complications, namely vascular trauma (phlebitis and infiltration) and occlusion. The secondary outcomes are related to the catheters (such as other removal causes, maintenance time, insertion attempts, and the number of catheters needed to achieve the prescribed intravenous treatment), but also explore patient’s satisfaction of the intravenous treatment received, as well as nurse’s satisfaction with the medical device (including the perception of safety/risk).

### Design and study setting

**Fig. 1:**
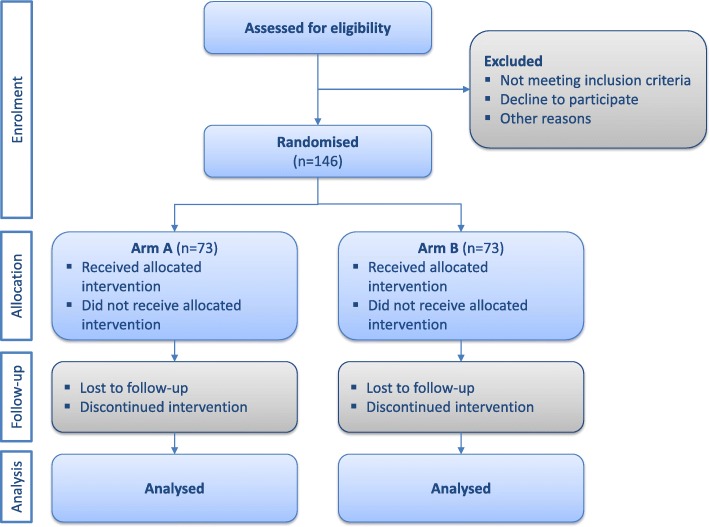
Flow diagram of the parallel randomised controlled trial with two groups (CONSORT 2010)

The study protocol was developed under the SPIRIT 2013 Guidelines [38]. The SPIRIT checklist is provided as Additional file 1. This is a multi-centre (three hospitals), two-arms, partially blinded randomised controlled trial (RCT; Fig. 1).

The principal investigator will obtain initial agreement from the three hospitals involved, and a lead investigator in each centre will be nominated, becoming the local contact person during the study. Before the clinical study starts, an investigator meeting will be conducted in each centre in order to present the clinical protocol and trial-specific procedures to the nurses’ research teams. The clinical study is planned to last 9 months from the start of the recruitment process to the reporting of results.

### Participant recruitment and eligibility criteria

The participants will be recruited in the orthopaedic departments of the three hospitals where the clinical study will take place. Prior to baseline data collection, eligible participants will be recruited by a research assistant nurse at the time of admission. Therefore, no plans will be made to increase the recruitment of participants across other clinical settings. Patients are admitted if they meet the following inclusion criteria:

- Patients aged 18 years or above, admitted to the orthopaedic department
- Patients with the ability to fully communicate in Portuguese
- Patients able to consent
- Prescribed PIVC for intravenous therapeutic administration
- PIVC expected to remain for at least 24 h
- PIVC inserted at the orthopaedic department
- PIVC size 18G or 20G
- Anatomical insertion site in arm, forearm, or back of the hand
- PIVC secured with a transparent, semi-permeable polyurethane film dressing

Patients will be excluded if they meet any of the following exclusion criteria:

- Patients with a known infectious disease
- Patients with leucocytosis, defined as ≥ 1200 leukocytes/mm^3^ [39]
- Patients with anaemia, with haemoglobin levels < 13 g/dl for men and < 12 g/dl for women [40]
- Patients receiving immunosuppressive treatment within 6 months prior to hospital admission
- Patients receiving chemotherapy or radiotherapy within 6 months prior to hospital admission
- Patients with body mass index below 16 kg/m^2^ or above 39 kg/m^2^ [22]
- Anatomical insertion site in flexion areas (e.g. cubital fossa region) or lower members
- Skin lesions at the insertion site (e.g. previous infiltration, dermatitis, burns) and skin alterations such as tattoos
- Peripheral venous alterations resulting from previous hospital admissions

### Randomization, concealment, and group allocation procedures

The participants will be randomised after meeting two eligibility criteria screenings (Table1). Eligible and consenting participants will be randomly assigned to one of two treatment groups (arm A, double-chamber syringe; arm B, traditional syringes) using simple randomization with a 1:1 ratio. In each participating hospital ward nurses will have access to a central randomization and online registration system (https://www.sealedenvelope.com/). Before patient inclusion in the study, ward nurses will log into the online system and provide the participant’s anonymous ID number. The central system will randomly allocate the participant to one of the two groups and inform the nurse in real time. All allocations will be recorded into the central system and can only be accessed by the study’s principal investigator, ensuring allocation concealment across sites.

**Table 1:**
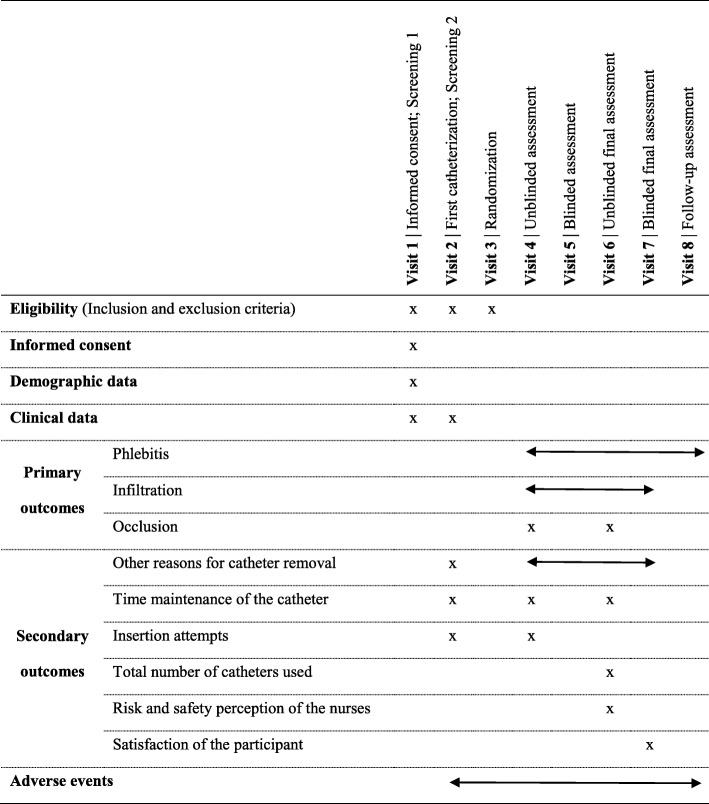
Assessment schedule for clinical study with the medical device

### Blinding

Patients and ward nurses will not be entirely blinded after group allocation due to the nature of the interventions. Despite this, participants will not be directly informed of their group allocation in terms of usual care or intervention, and the ward nurses will only be informed about the broad purposes of the research [41]—the study of a new double-chamber syringe for intravenous administration and flushing. In contrast, research nurses from the team outside the orthopaedic department will be blinded when rating some of the main outcomes such as phlebitis and infiltration. In accordance, external data analysts involved in the study will also be blinded to patient allocation and treatment groups.

### Interventions

The research/ward nurses will collect baseline personal (e.g. age, sex) and clinical data (e.g. intravenous medication prescribed, other medication, medical history). Data about the catheterization process includes date and time of catheter insertion, antiseptic used, catheter size, drugs injected, site of the insertion, dressings and devices used, skin assessment or vein visibility, as well as pain related to catheter insertion as reported by the patients. Ward nurses will receive a brief educational session focused on the flushing procedure during intravenous drug administrations and aseptic principles in order to ensure standardization of all the clinical procedures during the clinical research.

The study protocol ensures that there is no selection bias, i.e. the participants in both groups received the same treatments/care, with the exception of the manipulated variable. Thus, the two groups only differ in the syringes used to perform the intravenous medication administration and flushing procedures. In arm A, nurses use the double-chamber syringe and the traditional syringes will be used in arm B. Several aspects regarding the catheterization, medication administration, and monitoring of the PICV will be ensured to be equal in both groups and in agreement with Good Clinical Practices.

The catheterization in both groups will be done according to the standard procedures of the aseptic technique [17, 19, 30, 42]. In both arms, the decision to change the PIVC will be based on clinical criteria, such as completion of therapy, phlebitis, infiltration, occlusion, accidental removal, or suspected infection [16, 19, 23, 43–45]. In fact, several studies indicate that the routine replacement of PIVCs (using predetermined time frames, e.g. 72–96 h) does not show advantages when compared to clinically indicated replacements [44–46]. Previous to the administration of the intravenous medication, vein permeability will be assessed and the pulsatile (push–pause) delivery action will be used to optimize flush outcomes and minimize damage to the vein [31, 33, 36]. All medication safety precautions inherent to intravenous medications will be followed when administering flush solutions. Any deviations from the protocol will be recorded.

Outcome assessment visits will be performed on a daily basis, in accordance with each participant’s period of prescribed intravenous drug administration. Using the same procedures in both groups (warranting the internal validity of the study), two distinct approaches will be considered (Table 1): (i) *unblinded visits* (always, at the time of the intravenous administration)—due to the nature of the interventions with the medical device, the wards nurses are aware of the participant allocation; (ii) *blinded visits* (once a day, outside the routine periods for drug administration by the ward nurses)—the research nurses are not aware of participant allocation and therefore there is no risk of distorting outcome measurements.

The *unblinded visits* will be performed by the ward nurses when the intravenous medications are administered (Table 1, type 4 visits). At this moment, the outcomes assessed will be phlebitis [47], infiltration [48], occlusion, catheter and insertion site analysis (main outcomes), as well as other reasons for catheter removal, maintenance time of the catheter, insertion attempts, and total number of catheters used (secondary outcomes; if applicable, i.e. when there is a need for new catheterization). The *blinded visits* will be performed by a research nurse who evaluates some of the main outcomes, namely phlebitis, infiltration, and catheter and insertion site analysis (Table 1, type 5 visits).

The last unblinded visit for outcome assessment will be performed at the end of the intravenous therapy by the ward nurse that removes the PIVC (Table 1, type 6 visits). During this visit, all the outcomes will be assessed by the ward nurses, with the exception of the insertion attempts and patient satisfaction. After the completion of the intravenous therapy and PIVC removal (Table 1, type 7 visits), research nurses will conduct a blinded visit similar to the previous type 5 visits. However, research nurses will also assess patients’ satisfaction about the clinical treatment. Finally, a follow-up assessment will be done (48–72 h after visit number 7) for the main outcome phlebitis (Table 1, visit 8). Throughout the study period, regardless of the type of visit, all adverse events will be assessed and recorded.

### Outcomes

The main primary outcomes are the catheter-related complications, such as vascular trauma (phlebitis and infiltration) and occlusion. Specific scales for phlebitis [47] and infiltration [48] assessment will be used and data analysed in terms of mean scores, standard deviations, and proportions. The occlusion will be rated by the ward nurses on the participants’ case report forms (CRFs) and analysed as proportions.

The secondary outcomes involve other reasons for catheter removal (e.g. accidental dislodgment), maintenance time of the catheter, number of syringes and catheters used during the intravenous treatment, catheterization attempts, nurses’ perception about risk and safety, as well as the satisfaction of the participants. The reasons for catheter removal are reported on the CRF and treated as proportions. The maintenance time of the catheter, number of syringes and catheters used, and catheterization attempts are also identified on the CRF, being analysed through mean scores and standard deviations.

Ward nurses’ and patients’ satisfaction about the clinical treatment will be rated using questionnaires focused on essential risk and safety features, in a five-point Likert scale, developed and validated by the research team for this purpose. These scores will be analysed through mean scores and standard deviations.

### Sample size

Commonly, the sample size calculation is based on an expected incidence of the primary outcome. In our study, we have several primary outcomes regarding PIVC-related complications. Therefore, the sample size was calculated considering the lower prevalence value in Portuguese contexts for one of the primary outcomes (namely occlusion, with a 19% prevalence rate) [13, 28]. Also, the sample size determination was established using G*Power software, taking into consideration the type I (α) error level, the statistical power (type II error, β), and the standard deviation of the measurement for continuous outcomes. Fisher’s exact test comparing the proportions in two groups (*p* control = 0.19; *p* experimental = 0.05) are considered (Table 2). Ensuring a type I error of 5% and a statistical power of 80%, the estimated sample size is 146 participants (73 participants in each group).

**Table 2:** Sample size estimation

Input		Output	
Tail(s)	One	Sample size group 1	73
Proportion p1	0.19	Sample size group 2	73
Proportion p2	0.05	Total sample size	146
α err prob	0.05	Actual α	0.0197
Power (1-β err prob)	0.80		
Allocation ratio N2/N1	1		

Fisher’s exact test: Proportions, inequality, two independent groups

### Data collection, management, and analysis

Paper CRFs will be used in this clinical study to record all the information required from the protocol. Distinct CRFs will be used for the blinded and unblinded nurses. All CRF data are anonymized for subsequent analysis and reports/publications. According to this, study participants will be assigned a unique subject identification (ID) number. Study subject ID numbers will be used on all data collection instruments. The names of the participants on the consent forms will be stored separately from CRFs in locked cabinets and only accessible by named personnel.

Data to be collected include individual characteristics, demographics, and clinical information. No biological specimens will be collected. Data will be entered and analysed using the Statistical Package for the Social Sciences, version 24 (IBM SPSS Statistics 24, SPSS Inc., Chicago, IL, USA). Baseline characteristics of patients and catheters will be described by group. Means, standard deviations, frequencies, and percentages will be used as descriptive statistics. For each group, the number of participants who were randomly assigned will be analysed for the outcomes. Data analysis will be done using intention-to-treat principles [49]. Missing data will be handled using the last observation carried forward method, which uses the last available data for each trial participant, at the visit prior to withdrawal from the study, in the data analysis [50]. Specifically, for each primary and secondary outcome, the estimated effect size and its precision will be analysed. Baseline data for both groups will be observed to check for similarity in important variables. According to these results, variables with statistically significant differences between groups will be controlled in subsequent outcome analyses. The primary and secondary outcomes in the two groups will be examined to detect the effect of group allocation on each condition. Specifically, differences between continuous variables will be analysed using Student’s *t-*test. For skewed data, median values and interquartile ranges will be calculated and undergo nonparametric analysis using Mann-Whitney *U* test. Categorical variables will be compared by χ^2^ test or Fisher’s exact test, as appropriate. We deemed *p* values less than 0.05 to be significant (two-sided significance level of 5%). No additional analyses (e.g. interim analyses, planned subgroup analyses) will be performed.

To ensure the efficiency and quality of the above procedures, a Steering Committee and Data Management team will be created, composed of the trial coordinator and the lead investigators from each collaborating centre. These teams will review and supervise ongoing trial tasks on a monthly basis, or whenever a non-expected event is reported. The representatives of the Data Management team in each collaborating centre will conduct weekly data checks in order to ensure the accuracy and quality of the information collected. Given the nature and duration of the trial, no Data Monitoring Committee will be created [51].

### Adverse event management

Adverse events associated with participation in this clinical study (e.g. catheter dislodgment or removal) will be considered as study outcomes. All adverse events will be registered by the ward/research nurses through a specific section in the developed CRF, according to article 22° of Portuguese law n°21/2014, and communicated to a subcontracted Medical Vigilance Committee (as legally required for the clinical research with medical devices under article 22° of Portuguese law n°21/2014). The Medical Vigilance Committee will analyse the event and produce a written report in the subsequent 5 days, which will be presented to the national competent authorities. To achieve high quality monitoring and reporting of adverse events, the ward and research nurses will be trained in Good Clinical Practices prior to study conduction by the Medical Vigilance Committee.

### Ethical considerations and dissemination

Being a clinical study with a new medical device, all legal requirements will be met, considering the European Commission (2017) guidelines [52] approved by INFARMED (Portuguese National Authority of Drugs and Health Products) and also the International Organization for Standardization norms (ISO 141155: 2011 [53] and ISO 14971: 2012 [54]). Additionally, the study will be conducted in compliance with the protocol and the principles of Good Clinical Practices. Given the nature of this study, initial central ethical approval concerning the early stages of analysis and development of the double-chamber syringe has been confirmed by the Ethics Committee of the Health Sciences Research Unit: Nursing the Nursing School of Coimbra (reference approval number P608–8/2019) and the Ethics Committee of the Federal University of Viçosa (reference approval number 9929188.8.0000.5153). However, trial recruitment at the three collaborating hospitals will not commence until local ethics approval has been obtained.

Information concerning this clinical study (e.g. research purposes, implications, and study procedures) will be presented to the patient by a research nurse, before their inclusion in the study, in order to obtain written consent. The study protocol and all the templates used (informed consent form, CRFs, questionnaires) will be reviewed and approved by the relevant ethics committees. All ethical considerations will be strictly adhered to, including informed consent and voluntary participation, ensuring all legal aspects regarding privacy and confidentiality.

Upon completion of the trial, the CONSORT 2010 (Consolidated Standards of Reporting Trials) [55] guidelines will be used to report the data obtained. The datasets generated and/or analysed during the trial will not be publicly available, but will be accessible from the trial coordinator on reasonable request.

The results will be disseminated in national and international, open-access, peer-reviewed journals, as well as in national and international meetings in this scientific area. A publication policy will be ensured between partners, including ownership and use of the intellectual property and publication rights. Authorship eligibility will be considered for all researchers that display substantive contributions to the design, conduct, interpretation, and reporting of the clinical trial [56], following the recommendations of the International Committee of Medical Journal Editors [57].

## Discussion

Intravenous catheterization is the most common invasive procedure among hospitalized patients, which has been associated with several complications. In fact, the procedure is not risk-free and PIVC failure is a common problem with serious consequences for patients, their families, and the healthcare system. PIVC failure can be mechanical (e.g. infiltration, occlusion), vascular (e.g. phlebitis), or infectious. When the catheter can no longer be used for treatment due to associated complications, new catheterization is necessary, which can result in negative consequences for the patient, such as pain and anxiety, delays in treatment, and unnecessary exposure to the risks associated with multiple insertions. The prevention of catheter-related complications implies some essential precautions regarding catheterization practice and catheter surveillance [17]. This is particularly important due to the higher prevalence of nosocomial infections in Portugal [25, 26], as well as the substantial incidence of phlebitis, infiltration, and occlusion [5, 13, 28].

Flushing the catheter is considered the most important factor in preventing malfunction because the catheter patency is maintained. Correspondingly, this procedure also prevents some important complications such as phlebitis, occlusion, or catheter colonization [31, 37]. The flushing is included in specific guidelines regarding the intravenous administration of therapeutics [17, 30]. To accomplish this, nurses need to use two or more syringes to deliver the drug and the flush solution. Although some advances have been made in the development of prefilled flush syringes and double-chamber syringes, the existing models do not address current clinical and economic challenges.

According to this, the importance of developing medical devices to improve nurses’ performance regarding effectiveness and safety involved in intravenous therapeutics administration procedures is well established. This two-armed randomised controlled study was designed to demonstrate the clinical effectiveness and safety of this new medical device, comparing this double-chamber syringe with the traditional syringes generally used for intravenous administration of medications/fluids and flushing procedures. Effectively, the well-defined primary and secondary outcomes aim to evaluate the effectiveness and safety of this new double-chamber syringe in comparison with traditional syringes.

The rigorous eligibility criteria will ensure the control of important variables that could impact on the trial’s results. For example, catheter size and insertion site are important factors for catheter longevity. Although the use of larger diameter catheters significantly increases catheter longevity [58] and reduces the risk of accidental removals [15], larger sizes also predict the incidence of phlebitis and subsequent catheter failure [15, 59]. In light of the contradictory results from several studies about catheter size, we defined the use of 18 or 20 gauge catheters, which are the most used in the orthopaedic departments that will participate in the clinical study. Regarding the anatomical insertion site, it is well accepted that upper extremity veins are desirable for patient comfort and fewer complications [16, 17, 19, 37]. Also, several studies have shown that catheter placement in the forearm significantly increased their longevity [15, 16, 37, 58]. Catheters in the cubital fossa region have been associated with higher incidence of complications such as phlebitis or occlusion [15, 16, 37, 59]. Similarly, anatomical areas of flexion must be avoided due to the high risk of complications such as dislodgement and phlebitis [17]. According to the existing evidence, this trial will only include patients with a PIVC inserted in their arm, forearm, or back of the hand.

Despite the strengths of this protocol, some limitations should be pointed out, such as the impossibility to perform a totally blinded assessment for all the outcomes. In fact, only a select number of outcomes will be assessed by the blinded research nurses (e.g. phlebitis), given that the majority of the outlined outcomes can only be assessed at the exact moment of intravenous administration with the double-chamber or traditional syringes. Moreover, the study will be implemented within a specific context (orthopaedics departments), which may condition a broader analysis of the results obtained. To address this challenge, future studies should be done in other clinical contexts that involve intravenous therapy administration.

## Conclusions

The development of innovative medical devices should attend important clinical problems, in order to assist nurses’ performance on good clinical practices and reducing several difficulties in clinical practice. Despite this, well-structured RCTs are needed to ensure the effectiveness and safety of any new medical devices. After the completion of data collection and subsequent analysis, the findings from this trial will make a significant contribution that substantiates the advantages of the newly developed double-chamber syringe. It is expected that this new syringe will facilitate nurses’ adoption of good clinical practices during intravenous drug administration, such as flushing, decreasing the overall time of preparation/administration, and minimizing the number of catheter manipulations, as well as a reduction in PIVC-related complications.

### Trial status

Pre-clinical studies on this new medical device are being completed in order to submit the clinical research protocol to Portuguese legal entities. The trial is registered at ClinicalTrials.gov (NCT04046770). Protocol version 5 dated February 1st, 2019. At this time, the first wave of research participants are expected to be recruited by July 2020. Participant recruitment is expected to be completed by October 2021. We expect the main RCT results to be published at the end of 2021.

## Supplementary information

**Additional file 1.** SPIRIT 2013 Checklist: Recommended items to address in a clinical trial protocol and related documents*.

## Data Availability

Not applicable.

## Abbreviations

CONSORT: Consolidated Standards of Reporting Trials
CRF: Case report form
ECDC: European Centre for Diseases Control and Prevention
INFARMED: Portuguese National Authority of Drugs and Health Products
ISO: International Organization for Standardization norms
PIVC: Peripheral intravenous catheter
PPCIRA: Program for Prevention and Control of Infection and Resistance to Antimicrobials
RCT: Randomised controlled trial
SPIRIT: Standard Protocol Items: Recommendations for Interventional Trials
